# Supplementary material for: Patterns of Intron Gain and Loss in Fungi
Source: PLoS Biol. 2004 Nov 30;2(12):e422. doi: 10.1371/journal.pbio.0020422 (PMC532390; doi:10.1371/journal.pbio.0020422)
Supplement: Table S1 — Also available at http://genes.mit.edu/NielsenEtAl/. (4.3 MB ZIP). [file pbio.0020422.st001.zip › NielsenEtAl/html/200.html]

AN1971.1.NCU03482.1.MG03958.1.FG05150.1


```
 CLUSTAL W (1.82) Multiple Sequence Alignments - Introns Inserted


Sequence 1: NCU03482.1	458 aa
Sequence 2: MG03958.1	486 aa
Sequence 3: FG05150.1	458 aa
Sequence 4: AN1971.1	478 aa
Alignment Length: 505 aa
Number Identitical Residues: 350 aa
Alignment Score (without introns) 14349


MG03958.1 	MVQISEVKGNSRENRTAAHTHIKGLGLRSDGTADKQGSGFVGQQTARE0ACGVVVDLIRS
NCU03482.1	MVQISEVKGNSRDNRTAAHTHIKGLGLNSQGIAEKQASGFVGQTTARE~ACGVVVDLIKA
FG05150.1 	MVQISEVKGNKRDNRTAAHTHIKGLGLKSDGYAEKQAAGFVGQAAARE0SCGVVVDLIRA
AN1971.1  	MVQISEVKGNSRDNRTAAHTHIKGLGLRPDGTAEVSGDGWVGQAAARE0ACGVVVDLIKA
          	**********.*:**************..:* *: .. *:*** :*** :********::

MG03958.1 	HKMAGRAVLLAGGPGTGKTALALAISQELGTKIPFCPIVGSEIYSTEVKKTEVLMENFRR
NCU03482.1	HKMAGRGVLLAGGPGTGKTALALAISQELGTKIPFCPITGSEIYSTEVKKTEVLMENFRR
FG05150.1 	QKMAGRGVLLAGGPGTGKTALALAISQELGTKIPFCPIVGSEIYSAEVKKTEMLMENFRR
AN1971.1  	KKMAGRAVLLAGGPGTGKTALALAVSQELGTKVPFCPIVGSEIYSAEVKKTEALMENFRR
          	:*****.*****************:*******:*****.******:****** *******

MG03958.1 	AIG------------------LKVRETKEVYEGEVTELTPEEAENPLGGYGKTISTLLIG
NCU03482.1	AIG------------------LKVRETKEVYEGEVTELTPEEAENPLGGYGKTITTLLIG
FG05150.1 	AIG------------------LKVRETKEVYEGEVTELTPEEAENPLGGYGKTISTLLIG
AN1971.1  	AIGMEWLYDILGAQTLTSWLGLRVRETKEVYEGEVTELTPQEAENPLGGYGRTISHLIIG
          	*** .   .  .:.: ::  .*:*****************:**********:**: *:**

MG03958.1 	LKSAKGQKKLRLDPSIYEAIQKERVTVGDVIYIEANTGACKRVGRSDAYATEFDLEAEEY
NCU03482.1	LKSAKGQKKLRLDPSIYEAIQKERVTVGDVIYIEANTGACKRVGRSDAYATEFDLEAEEY
FG05150.1 	LKSAKGQKKLRLDPSIYEAIQKERVTVGDVIYIEANTGACKRVGRSDAYATEFDLEAEEY
AN1971.1  	LKSAKGTKKLRLDPSIYEAIQKERVTVGDVIYIEANTGACKRVGRSDAYATEFDLEAEEY
          	****** *****************************************************

MG03958.1 	VPIPKGEVHKKKEIVQDVTLHDLDVANARPQGGQDIMSMMGQLMKPKMTEITDKLRGEIN
NCU03482.1	VPIPKGEVHKKKEIVQDVSLHDLDVANARPQGGQDIMSMMGQLMKPKMTEITDKLRSEIN
FG05150.1 	VPIPKGEVHKKKEIVQDVSLHDLDVANSRPQGGQDIMSMMGQLMKPKMTEITDKLRGEIN
AN1971.1  	VPVPKGEVHKKKEIVQDVTLHDLDIANARPQGGQDVMSMMGQLMKPKKTEITDKLRQEIN
          	**:***************:*****:**:*******:*********** ******** ***

MG03958.1 	KVVSKYIDQGVAELVPGVLFIDE0AHMLDIECFTYLNRALESPISPIVVLASNRGMTKIR
NCU03482.1	KVVSKYIDQGVAELVPGVLFIDE0AHMLDVECFTYLNKALESPISPIVVLASNRGMTGIR
FG05150.1 	KVVSKYIDQGVAELVPGVLFIDE0AHMLDVECFTYLNRALESPIAPIVVLASNRGMCTIR
AN1971.1  	KVVNRYIDQGVAELVPGVLFIDE0VHMLDIECFTYLNRALESSISPIVILASNRGHTVIR
          	***.:****************** .****:*******:****.*:***:******   **

MG03958.1 	GTDDLVAAHGVPPDFLARLLIIPTTPYDAEEIKRIVKLRASTEGVAISEAAIEKIAEHGV
NCU03482.1	GAEDLVAAHGIPPDFLSRLLIIPTTAYDPEEIKRIVKIRSTTEGVKITEAAIDKIAEHGV
FG05150.1 	GTDDIVAAHGIPSDFLARMLIIPTTPYEADEIKRIVRIRSTTEGVSVSDAAIDKISEHGV
AN1971.1  	GTDDISAAHGIPPDLLARLLIIPTHPYSPDEIKTIIRLRAKTEGLNITDPALDKVAEHGS
          	*::*: ****:*.*:*:*:***** .*..:*** *:::*:.***: :::.*::*::*** 

MG03958.1 	RISLRYCLQLLYPSS2ILARVNGR-KEIDIKDVAECEDLFLDARRSASLLASGD~TASGF
NCU03482.1	RISLRYCLQLLTPAS2ILAKVNGR-NEIDVQDVAECEDLFLDARRSAALLSS-E~QGQEF
FG05150.1 	RISLRYCLQLLTPAS2ILAKANGR-SQIDVQDVAECEDLFLDASRSAALLSS-E~AGRGY
AN1971.1  	KVSLRYALQLLTPAS~ILARVNGRPGGIEEADVTECEDLFLDSKRSAAIVNQDS1EISGF
          	::****.**** *:* ***:.***.  *:  **:********: ***::: ...     :

MG03958.1 	I0IRRKLENKRNQTEGQFPIVQDVRQGEGK
NCU03482.1	I~C---------------------------
FG05150.1 	L~A---------------------------
AN1971.1  	S~P---------------------------
          	
```
